# Supplementary material for: Development of Imeglimin Electrospun Nanofibers as a Potential Buccal Antidiabetic Therapeutic Approach
Source: Pharmaceutics. 2023 Apr 11;15(4):1208. doi: 10.3390/pharmaceutics15041208 (PMC10144366; doi:10.3390/pharmaceutics15041208)
Supplement: Supplementary file 1 [file pharmaceutics-15-01208-s001.zip › pharmaceutics-2222882-supplementary.pdf]

Supplementary Materials Section

(1) The chemical structure of imeglimin hydrochloride.

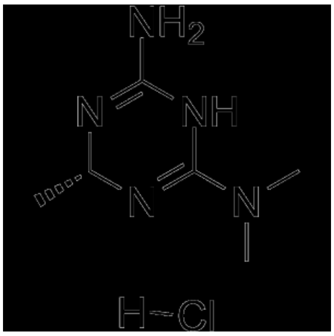

Figure S1. Imeglimin HCl structure. Mwt. 191.66; water solubility ≥ 50 mg/mL (260.88 mM); DMSO solubility: 25 mg/mL (130.44 mM; Need ultrasonic). [29]

(2) The successful separation of imeglimin by the developed HPLC method showed that the drug appeared at 3.3 min (i.e., Rt).

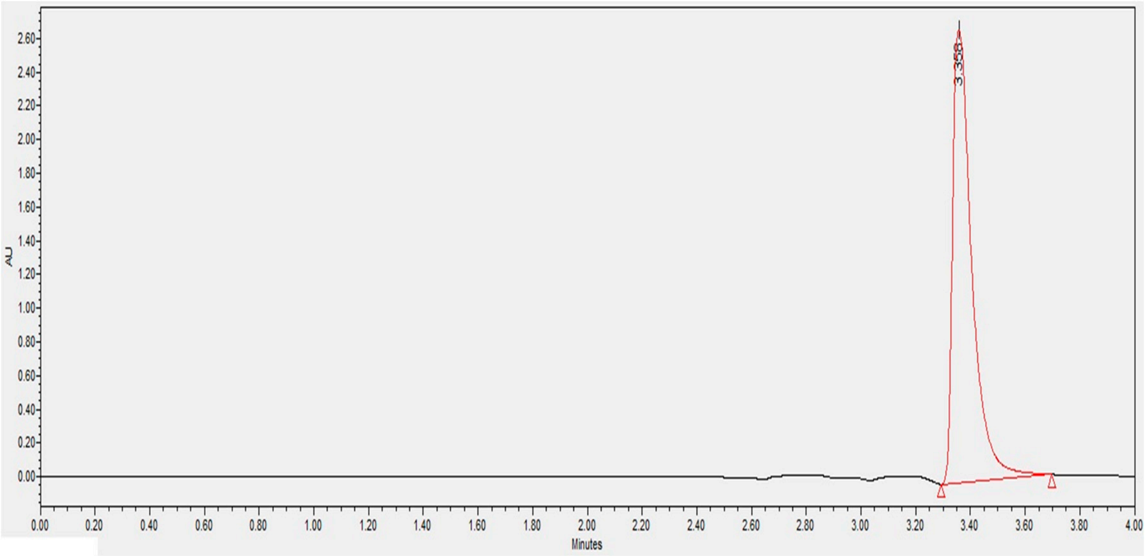

Figure S2. The developed HPLC method of imeglimin shows the successful separation of the drug at Rt 3.3 min. A 100 μg/mL drug concentration was used as a representative chromatogram.
